# Predicting clinical benefit response after neoadjuvant chemotherapy in locally advanced gallbladder cancer: retrospective analysis

**DOI:** 10.1093/bjsopen/zraf077

**Published:** 2025-08-12

**Authors:** Shraddha Patkar, Kaival Gundavda, Kaushik Polusany, Raghav Yelamanchi, Gurudutt P Varty, Niket Shah, Akash Pawar, Vikas Ostwal, Anant Ramaswamy, Prabhat Bhargava, Mahesh Goel

**Affiliations:** Department of Gastrointestinal and Hepatobiliary Surgery, Department of Surgical Oncology, Tata Memorial Hospital, Homi Bhabha National Institute (HBNI), Mumbai, India; Department of Gastrointestinal and Hepatobiliary Surgery, Department of Surgical Oncology, Tata Memorial Hospital, Homi Bhabha National Institute (HBNI), Mumbai, India; Department of Gastrointestinal and Hepatobiliary Surgery, Department of Surgical Oncology, Tata Memorial Hospital, Homi Bhabha National Institute (HBNI), Mumbai, India; Department of Gastrointestinal and Hepatobiliary Surgery, Department of Surgical Oncology, Tata Memorial Hospital, Homi Bhabha National Institute (HBNI), Mumbai, India; Department of Gastrointestinal and Hepatobiliary Surgery, Department of Surgical Oncology, Tata Memorial Hospital, Homi Bhabha National Institute (HBNI), Mumbai, India; Department of Gastrointestinal and Hepatobiliary Surgery, Department of Surgical Oncology, Tata Memorial Hospital, Homi Bhabha National Institute (HBNI), Mumbai, India; Department of Biostatistics, Tata Memorial Hospital, Homi Bhabha National Institute (HBNI), Mumbai, India; Department of Medical Oncology, Tata Memorial Hospital, Homi Bhabha National Institute (HBNI), Mumbai, India; Department of Medical Oncology, Tata Memorial Hospital, Homi Bhabha National Institute (HBNI), Mumbai, India; Department of Medical Oncology, Tata Memorial Hospital, Homi Bhabha National Institute (HBNI), Mumbai, India; Department of Gastrointestinal and Hepatobiliary Surgery, Department of Surgical Oncology, Tata Memorial Hospital, Homi Bhabha National Institute (HBNI), Mumbai, India

**Keywords:** GBC, CBR

## Abstract

**Background:**

Neoadjuvant chemotherapy is increasingly used in patients with locally advanced gallbladder cancer (LAGBC). This study investigated factors affecting clinical benefit response (CBR) to neoadjuvant chemotherapy for LAGBC.

**Methods:**

All consecutive patients with LAGBC following neoadjuvant chemotherapy, from January 2013 to December 2022, were analyzed for clinical and radiological responses as well as survival outcomes. CBR rates, resectability, and their impact on survival were evaluated. In addition, factors predicting CBR were identified and a predictive nomogram model was developed.

**Results:**

Of 401 patients with LAGBC undergoing neoadjuvant chemotherapy, 303 (75.5%) exhibited a CBR. The median overall survival (OS) in patients with a CBR was 25 months, compared with 8.5 months for those without a CBR. Factors predicting a worse CBR rate included age ≥ 55.5 years (hazard ratio (HR) 2.17; 95% confidence interval (c.i.) 1.29 to 3.65), Eastern Cooperative Oncology Group (ECOG) performance status ≥ 1 (HR 2.5; 95% c.i. 1.117 to 5.59), platelet count ≥ 468 × 10^9^/l (HR 2.86; 95% c.i. 1.12 to 6.74), tumour (T) size ≥ 2.1 cm (HR 3.4; 95% c.i. 1.70 to 6.80), T stage ≥ T3 (HR 3.26; 95% c.i. 1.22 to 8.74), and a systemic immune-inflammation index (SII) ≥ 1265.90 (HR 2.34; 95% c.i. 1.27 to 4.30). Of the patients with a CBR, 86% underwent curative surgical resection, with median OS improved to 29.54 months, compared with 11.86 months for those without resection (*P* < 0.01).

**Conclusion:**

A CBR was achieved in 75.5% of patients, with curative surgical resection in 86%. A CBR was associated with improved OS. Anatomical (T size, T stage) and immune–inflammation markers (platelet count, SII) were found to predict a CBR, and could help identify responders to neoadjuvant chemotherapy. This could have implications for treatment strategies, but requires validation in further prospective studies.

## Teaser

A clinically beneficial response (CBR) to neoadjuvant chemotherapy is associated with significantly improved survival. Factors associated with a worse CBR rate include age at diagnosis ≥ 55.5 years, Eastern Cooperative Oncology Group performance status ≥ 1, a platelet count ≥ 468 × 10^9^/l, tumour size ≥ 2.1 cm, T stage ≥ T3 and a systemic immune-inflammation index ≥ 1265.90. Patients with a score of > 25.2 on the nomogram developed to predict a CBR in this study are less likely to achieve a CBR.

## Introduction

Gallbladder cancer (GBC) is the most common biliary tract malignancy and is especially prevalent in Latin America and on the Indian subcontinent. Although surgical resection may offer a cure when GBC is detected early, over 80% of patients are diagnosed with advanced stage disease^1^. Treatment of locally advanced GBC (LAGBC) poses a dilemma, with ill-defined neoadjuvant treatment strategies, a lack of reliable indicators to predict tumour response, and poor long-term survival.

The Tata Memorial Hospital (TMH) Criteria identify patients at higher risk of disease recurrence and who are likely to benefit from neoadjuvant chemotherapy (NACT) (*Table S1*)^2^. Although there are insufficient data to support the routine use of NACT or neoadjuvant chemoradiation, the potential benefits of neoadjuvant therapy include tumour downstaging, early treatment of micrometastases, biological selection of patients before surgery, and a possible improvement in survival^3,4^.

Patients with a complete or partial response or with stable disease following NACT are considered to have clinical benefit response (CBR), which correlates with survival^2,5^. There remains an unmet need for reliable predictors of the tumour response to NACT.

Chronic inflammation is crucial in metaplasia-carcinoma transformation and tumour progression^1^. Inflammation-associated markers such as the neutrophil-to-lymphocyte ratio (NLR), platelet-to-lymphocyte ratio (PLR) and systemic immune-inflammation index (SII) serve as prognostic indices for survival in biliary tract cancers^6–8^. The Prognostic Nutritional Index (PNI) reflects immune–inflammatory and nutritional status and predicts chemotherapy response and survival^9^. In this study, the predictive value of pretreatment inflammatory, nutritional, and tumour-specific indices for predicting a CBR to NACT was evaluated.

## Methods

### Study population

Data were collected from a prospectively maintained database in the Gastrointestinal and Hepato-Biliary Division of the Department of Surgical Oncology at the Tata Memorial Hospital (Mumbai, India). Consecutive patients with LAGBC or borderline resectable GBC, defined per the TMH Criteria^2^, receiving NACT with curative intent between January 2013 and December 2022 were included in the study and analysed for clinicoradiological response and survival outcomes. Patients undergoing upfront gallbladder resection, curative or palliative resection for metastatic disease, and those with non-adenocarcinoma histologies (neuroendocrine tumours and adenosquamous tumours) were excluded.

### Primary assessment

The primary evaluation included a physical examination, ultrasound of the abdomen, contrast-enhanced cross-sectional imaging, and serum tumour markers (carcinoembryonic antigen and carbohydrate antigen 19.9). The preferred initial imaging modality was a triple-phase contrast-enhanced computed tomography (CECT) scan of the thorax, abdomen, and pelvis. ^18^-F-Fluorodeoxyglucose positron emission tomography/CECT was performed to rule out distant metastasis in patients with LAGBC or incidental GBC (iGBC) 4 weeks after the index surgery^10,11^. In patients without metastases, a laparoscopy was performed to rule out occult peritoneal disease. Histological diagnosis was obtained using percutaneous fine needle aspiration biopsy before initiating neoadjuvant systemic therapy. The pathology was confirmed in patients who had already undergone a biopsy or cholecystectomy elsewhere.

### Baseline assessment of inflammatory and nutritional markers

Markers of inflammation, namely NLR, PLR, SII, and the pan-immune-inflammation value (PIIV), were recorded before treatment initiation. SII, PIIV, and PNI were calculated using the following formulas:


SII=P×N/L

PIIV=N×P×M/L

PNI=(10×serumalbumin(g/d1))+(0.005×L)

where P, N, M, and L are pretreatment peripheral blood platelet, neutrophil, monocyte, and lymphocyte counts.

### Treatment schedule

Patients with radiologically suspected early GBC underwent upfront surgery^12^, whereas patients who fulfilled the TMH Criteria were treated with NACT or neoadjuvant chemoradiotherapy^2,13^. NACT comprised either a gemcitabine–cisplatin combination (gemcitabine 1000 mg/m^2^ as a 30-min infusion on days 1 and 8 and cisplatin 25 mg/m^2^ on days 1 and 8 of a 21-day cycle) or gemcitabine–oxaliplatin (gemcitabine 1000 mg/m^2^ on day 1 as a 100-min infusion and oxaliplatin 100 mg/m^2^ on day 2 over 2 h every 14 days). In some patients, nab-paclitaxel was added to the gemcitabine–cisplatin combination.

### Definition of responses and survival

Response to treatment was assessed with a CECT or PET/CECT scan after three to four cycles of NACT, and reported as per the RECIST criteria (Version 1.1)^14^.

The CBR was used as the endpoint because it assessed both objective response and disease stabilization rates. In this study, the CBR rate was defined as the percentage of patients who achieved a complete response or partial response or had stable disease following neoadjuvant therapy^15^. The objective response rate (ORR) was defined as the percentage of patients achieving a complete response or partial response to neoadjuvant therapy^15^. Patients with a CBR were considered for surgical resection 4 weeks after completion of NACT provided a margin-negative resection was deemed feasible on response imaging. Patients with disease progression, major vascular involvement (> 180°) and the involvement of two or more extrahepatic organs were considered unresectable. These patients received further chemotherapy, with or without radiation therapy, with a palliative intent.

### Pathology reporting

Among patients who underwent curative surgical resection, histopathology included assessment of tumour location, tumour size, histological differentiation, lymphovascular invasion, perineural invasion, treatment-related changes, and node involvement. Pathological staging was based on the American Joint Committee on Cancer^16^. Patients who underwent surgery received adjuvant chemotherapy to complete 6 months of perioperative therapy and were subsequently followed up as per the National Comprehensive Cancer Network recommendations^17^.

### Statistical analysis

Overall survival (OS) was calculated as the time elapsed between the date of diagnosis (date of registration, if the diagnosis was established before presenting to the institute) and death or date of last contact. Disease-free survival (DFS) was calculated from the date of curative surgery to the date of first recurrence. In unresected patients, the endpoint was considered as progression-free survival. Descriptive statistics were used to summarize the data. Univariable analysis was conducted to examine the relationship between each predictor variable and CBR. Multivariable logistic regression analysis was performed to build the multivariable model. Model fit was assessed using goodness-of-fit tests and receiver operating characteristic (ROC) curves. The ROC method was used to obtain the optimal cut-off values for biomarkers to predict a CBR. Regression coefficients and odds ratios (ORs) for each predictor variable in the final logistic regression model were calculated. Weighted scoring points were assigned to each predictor based on their coefficients. Raw scores were transformed into a probability score using logistic transformation. Nomogram performance was validated using measures of discrimination (for example, ROC curves and area under the ROC curve (AUC)). The Kaplan–Meier method was used to plot survival curves and survival estimates for OS and DFS.

The cohort was divided into two groups, one with a high probability of a CBR and the other with a low probability of a CBR, based on the median of the total nomogram score. Log-rank tests were used to compare survival between two independent groups. The Cox proportional hazards model was used to estimate the hazard ratio (HR) for the subgroup that underwent curative surgery adjusted for estimated scores for NACT among the responders. Two sided *P* < 0.050 was considered statistically significant. The model's c-statistic or Harrell’s C-index with 95% confidence interval (c.i.) was estimated using the bootstrap method to compare the unadjusted and adjusted models. All analyses were performed using SPSS^®^ version 26 (IBM, Armonk, NY, USA) and R software for statistical computing version 4.3 (R Foundation for Statistical Computing, Vienna, Austria).

## Results

### Patient demographic information and baseline characteristics

In all, 401 consecutive patients with locally advanced/borderline resectable gallbladder adenocarcinoma undergoing NACT between January 2013 and December 2022 were identified. Of these patients, 139 were diagnosed with iGBC and 262 had *de-novo* LAGBC (*Table 1*). Of the 401 patients undergoing NACT, 363 (90%) received a gemcitabine–platinum-based two-drug combination regimen, with cisplatin administered to 298 patients (74%) and oxaliplatin administered to 65 patients (16.2%). The median number of chemotherapy cycles was 3 (range 1–9), with 296 patients (73.8%) receiving three or four cycles as in the neoadjuvant setting. In all, 303 achieved a CBR (CBR rate = 75.56%), whereas the ORR was 55.86% (224 of 401 patients). The median follow-up was 60 (interquartile range (i.q.r.) 55–68) months (*Tables 2*, *3*).

**Table 1:** Tata Memorial Hospital Criteria for locally advanced/borderline resectable GBC used as an indication for neoadjuvant chemotherapy

Tumour characteristics		No. of patients
**Per primum GBC (*n* = 262)**		
Tumour (T3–T4 tumours)	Contiguous liver involvement > 2 cm	127 (31.6%)
	Involvement of bile duct causing OJ (type I/II block on MRCP/ERCP/PTBD)	29 (7.23%)
	Radiological/endoscopic involvement of adjacent viscera	24 (5.98%)
Node (N1 station)	Radiological suspicion of lymph node involvement N1	61 (15.2%)
Vascular (T4 tumours)	Impingement/involvement (< 180° angle) of one or more of hepatic artery and portal vein	21 (5.23%)
For incidental GBC (*n =* 139)	Residual/recurrent mass in gallbladder fossa/liver bed	91 (22.7%)
	N1 nodes as per nodal criteria	42 (10.47%)
	Involvement of bile duct causing OJ (type I/II block)	06 (1.5%)
Total no. of patients		401

GBC, gallbladder cancer; T, tumour stage; OJ, obstructive jaundice; N, nodal stage; MRCP, magnetic resonance cholangiopancreatography; ERCP, endoscopic retrograde cholangiopancreatography; PTBD, percutaneous transhepatic biliary drainage; N, nodal stage.

**Table 2:** Comparison of demographic and tumour characteristics of all patients and in those with and without CBR separately

	All patients (*n* = 401)	CBR (*n* = 303)	No CBR (*n* = 98)	P*
**Demographics**				
Age at diagnosis				
< 55.5 years	190 (47.4%)	157 (51.8%)	33 (33.7%)	
≥ 55.5 years	211 (52.6%)	146 (48.2%)	65 (66.3%)	0.002
Sex				
Male	138 (34.4%)	106 (35%)	32 (32.7%)	
Female	263 (65.6%)	197 (65%)	66 (67.3%)	0.764
ECOG performance status				
0	66 (16.5%)	57 (18.8%)	9 (9.2%)	
≥ 1	335 (83.5%)	246 (81.2%)	89 (90.8%)	0.037
Jaundice				
Yes	82 (20.4%)	54 (17.8%)	28 (28.6%)	
No	319 (79.6%)	249 (82.2%)	70 (71.4%)	0.022
Bilirubin				
< 6.58 mg/dl	345 (89.1%)	271 (92.2%)	74 (79.6%)	
≥ 6.58 mg/dl	42 (10.9%)	23 (7.8%)	19 (20.4%)	0.001
**Inflammatory and nutritional indices**				
Absolute neutrophil count				
< 7.15 × 10^9^/l	330 (82.3%)	264 (87.1%)	66 (67.3%)	0.000
≥ 7.1 × 10^9^/l	71 (17.7%)	39 (12.9%)	32 (32.7%)	
Absolute lymphocyte count				
< 5.15 × 10^9^/l	399 (99.5%)	302 (99.7%)	97 (99%)	0.399
≥ 5.15 × 10^9^/l	2 (0.5%)	1 (0.3%)	1 (1%)	
Absolute monocyte count				
< 0.49 × 10^9^/l	211 (52.6%)	165 (54.5%)	46 (46.9%)	0.195
≥ 0.49 × 10^9^/l	190 (47.4%)	138 (45.5%)	52 (53.1%)	
TLC				
< 11.55 × 10^9^/l	354 (88.3%)	278 (91.7%)	76 (77.6%)	
≥ 11.55 × 10^9^/l	47 (11.7%)	25 (8.3%)	22 (22.4%)	0.000
Platelet count				
< 468 × 10^9^/l	363 (90.5%)	284 (93.7%)	79 (80.6%)	
≥ 468 × 10^9^/l	38 (9.5%)	19 (6.3%)	19 (19.4%)	0.000
NLR				
< 3.19	266 (66.3%)	220 (72.6%)	46 (46.9%)	
≥ 3.19	135 (33.7%)	83 (27.4%)	52 (53.1%)	0.000
PLR				
< 162	224 (55.9%)	185 (61.1%)	39 (39.8%)	
≥ 162	177 (44.1%)	118 (38.9%)	59 (60.2%)	0.000
NLR : PLR				
< 0.0379	374 (93.3%)	289 (95.4%)	85 (86.7%)	
≥ 0.0379	27 (6.7%)	14 (4.6%)	13 (13.3%)	0.003
PNI				
< 61.2	353 (88%)	277 (91.4%)	76 (77.6%)	
≥ 61.2	48 (12%)	26 (8.6%)	22 (22.4%)	0.001
SII				
< 1265.9	315 (78.6%)	257 (84.8%)	58 (59.2%)	
≥ 1265.9	86 (21.4%)	46 (15.2%)	40 (40.8%)	0.000
PIIV				
< 395	240 (59.9%)	202 (66.7%)	38 (38.8%)	
≥ 395	161 (40.1%)	101 (33.3%)	60 (61.2%)	0.000
**Tumour characteristics**				
T size				
≤ 2.1 cm	172 (42.9%)	157 (51.8%)	15 (15.3%)	
> 2.1 cm	229 (57.1%)	146 (48.2%)	83 (84.7%)	0.000
Clinical N stage				
N0	152 (37.9%)	126 (41.6%)	26 (26.5%)	
N1	249 (62.1%)	177 (58.4%)	72 (73.5%)	0.008
Clinical T stage				
≤ T2	122 (58.4%)	97 (67.4%)	25 (38.5%)	
T3, T4	87 (41.6%)	47 (32.6%)	40 (61.5%)	0.000

Values are *n* (%). CBR, clinical benefit response; ECOG, Eastern Cooperative Oncology Group; TLC, total leucocyte count; NLR, neutrophil : lymphocyte ratio; PLR, platelet : lymphocyte ratio; PNI, Prognostic Nutrition Index; SII, systemic immune-inflammation index; PIIV, pan-immune-inflammation value; T, tumour stage; N, nodal stage. *Chi-square test.

**Table 3:** Treatment characteristics in all patients and in those with and without CBR separately

Treatment characteristics	All patients (*n* = 401)	CBR (*n* = 303)	No CBR (*n* = 98)	P*
**NACT regimen**				
Gemcitabine–cisplatin	298 (74.3%)	211 (69.6%)	87 (88.8%)	
Gemcitabine–oxaliplatin	65 (16.2%)	61 (20.1%)	4 (4.1%)	0.000
Gemcitabine–cisplatin–nab paclitaxel	30 (7.5%)	23 (7.6%)	7 (7.1%)	
Others	8 (2%)	8 (2.6%)	0	
**Treatment response**				
Partial response	120 (29.9%)	120 (39.6%)	0	
Complete response	104 (25.9%)	104 (34.3%)	0	0.000
Stable disease	79 (19.7%)	79 (26.1%)	0	
Progressive disease	98 (24.4%)	0	98 (100%)	
**Curative surgical resection**				
Yes	231 (57.6%)	228 (75.2%)	3 (3.1%)	
No	170 (36.9%)	75 (24.8%)	95 (96.9%)	0.000
**Extent of surgical resection**				
Radical cholecystectomy	82 (35.5%)	80 (35.1%)	2 (66.7%)	
+ EHBTE	21 (9.1%)	21 (9.2%)		0.001
+ adjacent organ resection	15 (6.5%)	14 (6.1%)	1 (33.3%)	
Revision cholecystectomy	108 (26.9%)	108 (47.4%)		
+ EHBTE	4 (1%)	4 (1.8%)		
+ adjacent organ resection	1 (0.2%)	1 (0.4%)		

Values are *n* (%). CBR, clinical benefit response; NACT, neoadjuvant chemotherapy; EHBTE, extrahepatic biliary tract excision. *Chi-square test.

The median OS of the entire cohort was 17.12 months, with a 5-year OS of 24% (20–29%). The median DFS was 12.7 months with a 5-year DFS of 21.7% (18–27%).

### CBR as a surrogate for survival

**Fig. 1:**
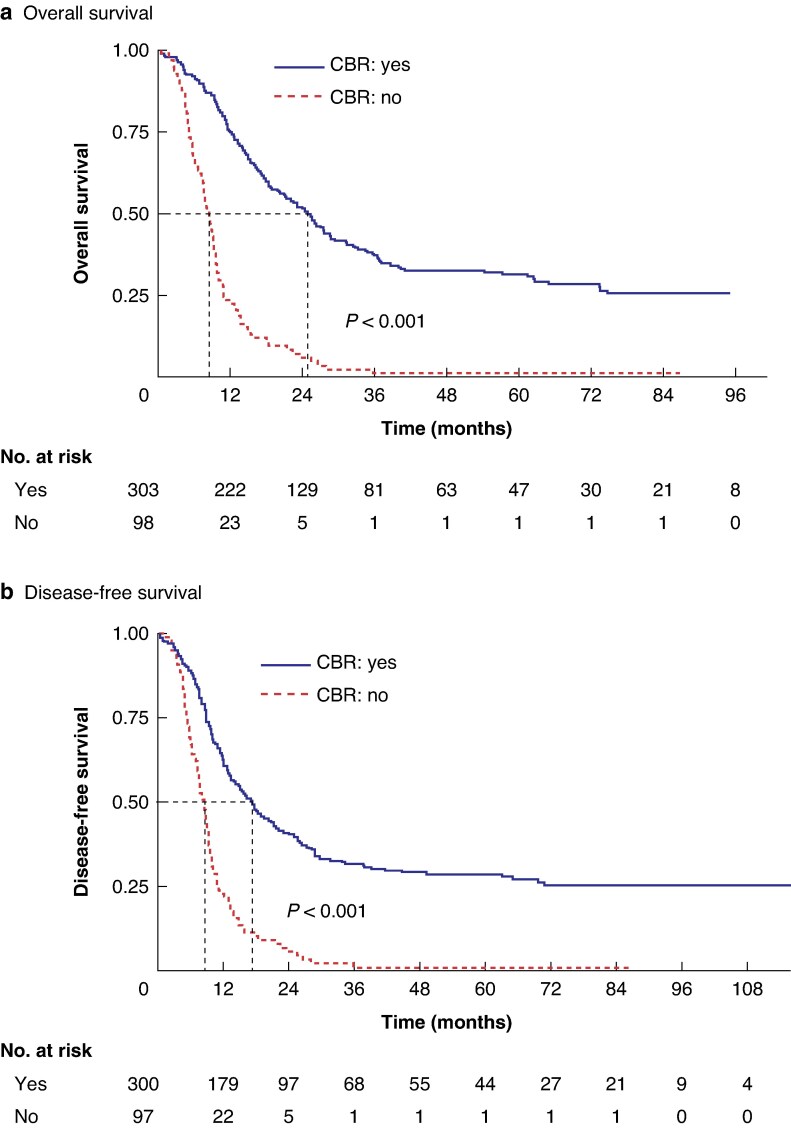
Kaplan–Meier curves showing a overall survival and b disease-free survival for patients with and without a CBR CBR, clinical benefit response.

A CBR was independently associated with significantly improved survival (*Fig. 1a*). The median OS for patients achieving a CBR was 25 (i.q.r. 21.2–28.5) months, compared with 8.5 (i.q.r. 7.6–9.3) months for those without CBR. The estimated median DFS for patients achieving a CBR was 17.3 (i.q.r. 14.4–21.3) months, compared with 5.58 (i.q.r. 7.46–9.36) months for those without a CBR.

### Predicting CBR

Age at diagnosis < 55.5 years, Eastern Cooperative Oncology Group (ECOG) performance status 0, the absence of jaundice at presentation, and lower levels of systemic inflammatory markers (NLR, PLR, PNI, SII, and PIIV) were associated with an improved CBR rate. Factors associated with a worse CBR rate included advanced T and N stage, tumour size > 2.1 cm, platelet count ≥ 468 × 10^9^/l, and higher metabolic activity of the primary tumour (maximum standardized uptake value > 11.6) (*Table 4*).

**Table 4:** Factors predicting a clinical benefit response

	Univariate		Multivariate	
	OR	*P*	OR	*P*
**Age at diagnosis**				
< 55.5 years				
≥ 55.5 years	2.11 (1.31, 3.40)	0.0020	2.17 (1.29, 3.65)	0.003
**Sex**				
Male				
Female	1.1 (0.68, 1.80)	0.6730	1.3 (0.59, 1.90)	0.59
**ECOG performance status**				
0				
≥ 1	2.29 (1.09, 4.82)	0.029	2.5 (1.117, 5.59)	0.026
**Jaundice**				
Yes				
No	0.542 (0.32, 0.92)	0.008	0.56 (0.32, 0.98)	0.09
**Bilirubin**				
< 6.58 mg/dl				
≥ 6.58 mg/dl	3.02 (1.56, 5.85)	0.001	3.4 (1.6, 5.97)	0.14
**Absolute neutrophil count**				
< 7.15 × 10^9^/l				
≥ 7.15 × 10^9^/l	3.28 (1.91, 5.63)	0.000	0.948 (0.343, 2.62)	0.918
**Absolute lymphocyte count**				
< 5.15 × 10^9^/l				
≥ 5.15 × 10^9^/l	3.11 (0.192, 50.24)	0.42	3.08 (0.2, 49.45)	0.319
**Absolute monocyte count**				
< 0.49 × 10^9^/l				
≥ 0.49 × 10^9^/l	1.35 (0.86, 2.13)	0.19	1.56 (0.9, 2.09)	0.2
**Total leucocyte count**				
< 11.55 × 10^9^/l				
≥ 11.55 × 10^9^/l	3.22 (1.7, 6.02)	0.0003	3.43 (1.6, 6.2)	0.07
**Platelet count**				
< 468 × 10^9^/l				
≥ 468 × 10^9^/l	3.59 (1.81, 7.11)	0.0002	2.86 (1.12, 6.74)	0.016
**Albumin**				
< 2.55 g/dl				
≥ 2.55 g/dl	0.6 (0.11, 3.50)	0.61	0.6 (0.10, 3.50)	0.62
**NLR**				
< 3.19				
≥ 3.19	2.99 (1.87, 4.79)	0.000	1.24 (0.52, 2.87)	0.60
**PLR**				
< 162				
≥ 162	2.37 (1.488, 3.77)	0.0003	1 (0.44, 2.25)	0.995
**NLR : PLR**				
< 0.0379				
≥ 0.0379	3.14 (1.42, 6.97)	0.0045	2.95 (1.4, 5.98)	0.067
**Prognostic Nutritional Index**				
< 61.2				
≥ 61.2	3.08 (1.65, 5.74)	0.0004	3 (1.5, 4.23)	0.19
**SII**				
< 1265.9				
≥ 1265.9	3.85 (2.312, 6.42)	0.000	2.34 (1.27, 4.3)	0.006
**PIIV**				
< 395				
≥ 395	3.15 (1.97, 5.06)	0.000	1.388 (0.596, 3.23)	0.447
**T size**				
≤ 2.1 cm				
> 2.1 cm	5.95 (3.28, 10.78)	0.000	3.4 (1.7, 6.8)	0.001
**Clinical T stage**				
≤T2				
T3, T4	6.79 (2.87, 16.06)	0.000	3.26 (1.22, 8.74)	0.018
**Clinical N stage**				
N0				
N1	1.97 (1.19, 3.26)	0.0082	2.4 (1.1, 4.1)	0.09
**PET SUV_max_**				
< 11.6				
≥ 11.6	3.30 (1.79, 6.07)	0.0001	2.47 (1.9, 6.2)	0.067

Values in parentheses are 95% confidence intervals. OR, odds ratio; ECOG, Eastern Cooperative Oncology Group; NLR, neutrophil : lymphocyte ratio; PLR, platelet : lymphocyte ratio; SII, systemic immune-inflammation index; PIIV, pan-immune-inflammation value; T, tumour stage; N, nodal stage; PET, positron emission tomography; SUV_max_, maximum standardized uptake value.

On multivariable analysis, age ≥ 55.5 years (HR 2.17; 95% c.i. 1.29 to 3.65; *P* = 0.003), ECOG performance status ≥ 1 (HR 2.5; 95% c.i. 1.117 to 5.59; *P* = 0.026), platelet count ≥ 468 × 10^9^/l (HR 2.86; 95% c.i. 1.12 to 6.74; *P* = 0.016), tumour size ≥ 2.1 cm (HR 3.4; 95% c.i. 1.70 to 6.80; *P* = 0.001), T stage ≥ T3 (HR 3.26; 95% c.i. 1.22 to 8.74; *P* = 0.018), and SII ≥ 1265.90 (HR 2.34; 95% c.i. 1.27 to 4.30; *P* = 0.006) were predictors of a worse CBR rate (*Table 4*).

**Fig. 2:**
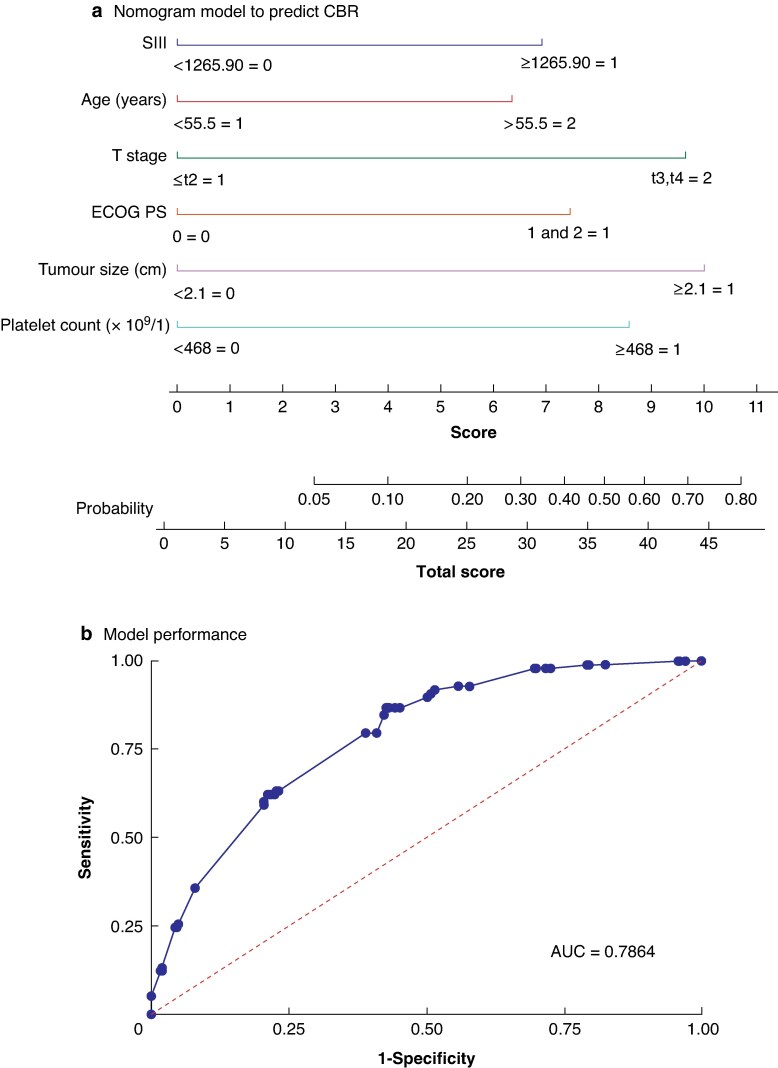
a Nomogram model for preoperative estimation of the probability of achieving a CBR and b performance of the model CBR, clinical benefit response; ECOG PS, Eastern Cooperative Oncology Group performance status; SII, systemic immune-inflammation index; AUC, area under the curve.

**Fig. 3:**
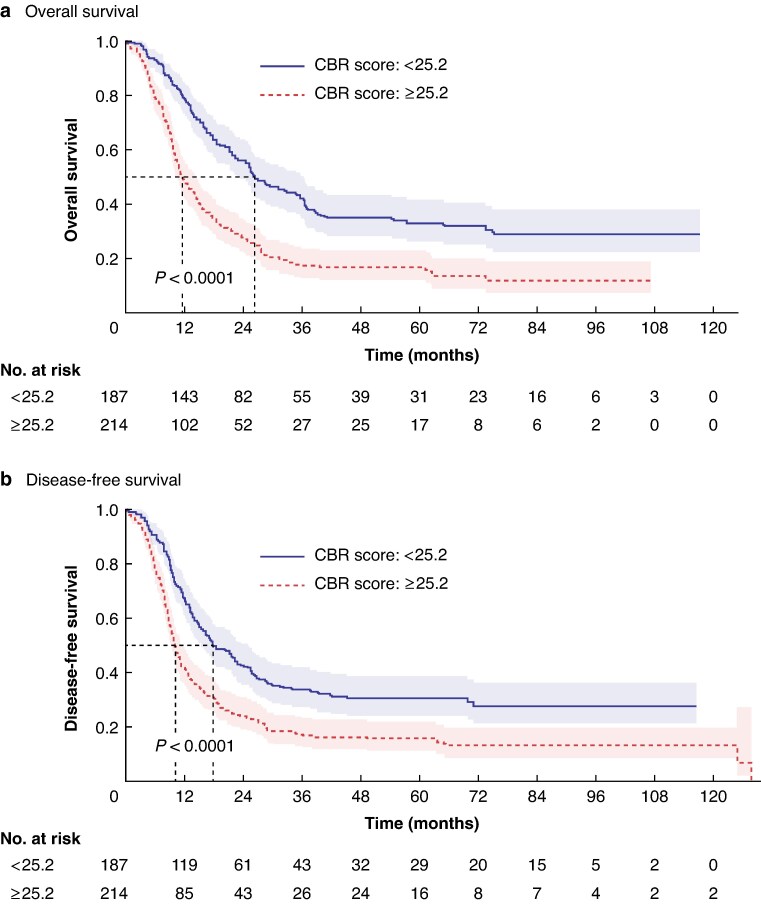
Kaplan–Meier curves showing a overall survival and b disease-free survival for patients assumed to have a low probability and high probability of a CBR based on a nomogram cut-off score of 25.2 Shaded areas indicate 95% confidence intervals. CBR, clinical benefit response.

These independent factors were integrated into a nomogram prediction model to indicate the probability of achieving a CBR, with the points allocated to each factor being calculated by the weight of their OR. ROC curve analysis revealed that the AUC of the nomogram model for predicting a CBR was 78.6% (*Fig. 2*). The optimal cut-off value was identified as 25.2, and was used to create two groups, one with a high probability of a CBR (187 patients) and the other with a low probability of a CBR (214 patients). Patients in the group with a high probability of a CBR had better OS (*Fig. 3a*) and DFS (*Fig. 3b*) than the patients in the group with a low probability of a CBR.

### Resectability

Of the 303 patients with a CBR, 289 (95.4%) underwent surgical exploration, with 228 (78.9%) subsequently undergoing curative surgery. Among these patients, margin-negative resection on pathology was achieved in 205 (90%). Of the 98 patients without a CBR, only 13 (13.3%) were offered surgical exploration, with only 3 of these patients deemed resectable (*Fig. S1*).

Within the cohort of patients with a CBR, the median OS was significantly improved in patients who underwent curative surgical resection than in those who did not (median 29.54 (i.q.r. 22.9–36.1) *versus* 11.86 (i.q.r. 8.42–15.3) months, respectively; *P* < 0.01). The median DFS of resected patients in the cohort with a CBR was 20.3 (i.q.r. 16.7–27.2) months, compared with 11.7 (i.q.r. 9.56–15.3) months for unresected patients (*P* < 0.001). However, the survival of unresected patients with a CBR was significantly better than that of patients without a CBR (median OS 8.57 months; *P* < 0.002).

In patients with a CBR, the unadjusted HR for OS for an estimated CBR prediction score ≥ 25.2 was 1.54 (95% c.i. 1.162 to 2.05; *P* = 0.003). After adjusting for curative surgery, the adjusted HR for OS for patients with an estimated CBR prediction score ≥ 25.2 decreased to 1.384 (95% c.i. 1.038 to 1.845; *P* = 0.027). The unadjusted HR (95% c.i.) for DFS for an estimated CBR prediction score ≥ 25.2 was 1.362 (95% c.i. 1.031 to 1.799; *P* < 0.03). After adjusting for curative surgery, the adjusted HR for DFS for patients decreased to 1.254 (95% c.i. 0.946 to 1.662), with *P* = 0.115.

## Discussion

This study is the largest evaluation of NACT in LAGBC or borderline resectable GBC, assessing CBR and its impact on long-term survival, while identifying novel inflammatory, clinical, and radiological predictive markers of a CBR. In this study, the CBR rate was 75.56% and the ORR was 55.86%. Previous studies, often on small, single-centre studies^2,18–24^, have reported CBR rates ranging from 48.8 to 100%, with ORR ranging between 14.4 and 100%. Systematic reviews have reported a CBR rate of 66–67%^3,4^ after NACT^2,18,19,21,22^ or neoadjuvant chemoradiation^20,23,24^.

Inflammation-associated markers, such as platelet count, NLR, and PLR, used as prognostic indices in various cancers^25–30^ have recently been recognized for biliary tract malignancies^6–8^. SII was initially noted for its prognostic value in patients with hepatocellular carcinoma after resection^30^. It is hypothesized that an elevated SII results in tumour dissemination, allowing cells to escape immune surveillance. Higher NLR, PLR, platelet count, and SII are associated with worse OS in GBC^31^. Although most studies have evaluated the association of preoperative levels of these markers with post-resection survival^32–34^, few have assessed their ability to predict responses to NACT. The PNI, which considers immune–inflammatory and nutritional status, has been shown to predict response to first-line chemotherapy in advanced biliary tract cancers^9^. The findings of the present study indicated that a platelet count ≥ 468 × 10^9^/l (HR 2.86; 95% c.i. 1.12 to 6.74; *P =* 0.016) and SII ≥ 1265.90 (HR 2.34; 95% c.i. 1.27 to 4.30; *P =* 0.006) could predict the lack of a CBR.

Historically, 40% (13.5–66.7%) of NACT patients achieved curative resection^3,4^; in the present study, 75.3% (302 of 401) of patients underwent exploration, with 231 patients finally resected. Of those patients who achieved a CBR, surgery was considered feasible in 95.4% based on response imaging, with curative resection possible in 78.9%. These higher rates of curative resection probably reflect the well defined clinicoradiological inclusion criteria, hence allowing for an accurate interpretation of the results.

The proposed nomogram stratified patients based on their likelihood of achieving a CBR, with good discriminative ability using clinical parameters, inflammatory markers, tumour size, and stage. By assigning an individualised probability score, clinicians could identify high-risk patients early and propose more aggressive and personalized treatments. Clinicians could optimize surgical candidacy, giving priority to surgical exploration, thanks to the fact that a high CBR could improve survival outcome.

Patients with high nomogram scores could benefit from intensified adjuvant strategies, as well as closer surveillance as a part of a risk-adaptive follow-up strategy. Molecular biological and genetic studies on patients who achieve a CBR could help identify sensitive targets for chemotherapy, as well as prognostic factors for GBC.

This study represents the largest experience of NACT use in LAGBC from a single high-volume centre, ensuring accurate results through defined clinicoradiological criteria. Study limitations include its retrospective design, which prevents standardization of NACT regimens, data on chemotoxicity, and dose reductions, as well as the inability to externally validate the proposed nomogram. The study population comprised patients from a single centre in India, which may limit the generalizability of the findings due to potential biological differences in tumour behaviour.

## Conclusion

Age at diagnosis ≥ 55.5 years, ECOG performance status ≥ 1, a platelet count ≥ 468 × 10^9^/l, tumour size ≥ 2.1 cm, T stage ≥ T3, and an SII ≥ 1265.90 predict poor CBR rates. This study highlights the significance of a CBR as an outcome measure following NACT, as well as a surrogate endpoint for survival. Future studies should explore the role of chemotherapy intensification for patients who are poor responders and the potential of immunotherapy and targeted agents. With further validation, the nomogram could become an integral part of risk assessment protocols, improving individualised patient care.

## Supplementary Material

zraf077_Supplementary_Data

## Data Availability

The data sets generated and analysed in the present study are available from the corresponding author upon reasonable request.
