# Supplementary material for: Predicting clinical benefit response after neoadjuvant chemotherapy in locally advanced gallbladder cancer: retrospective analysis
Source: BJS Open. 2025 Aug 12;9(4):zraf077. doi: 10.1093/bjsopen/zraf077 (PMC12343121; doi:10.1093/bjsopen/zraf077)
Supplement: zraf077_Supplementary_Data [file zraf077_supplementary_data.docx]

**Title:**

Predicting Clinical Benefit Rate (CBR) after neoadjuvant chemotherapy in locally advanced gallbladder cancer: retrospective analysis

**Author names and affiliations:**

*****Shraddha Patkar^a^ , MCh, *****Kaival Gundavda^a^ , MCh, Kaushik Polusany^a^ , MCh, Raghav Yelamanchi^a^ , MS, Gurudutt P. Varty^a^, MCh, Niket Shah^a^, MCh, Akash Pawar^b^, MSc, Vikas Ostwal^c^, DM, Anant Ramaswamy^c^, DM, Prabhat Bhargava^c^, DM, Mahesh Goel^a^, MS

***Contributed equally to this work as first authors**

^a^Department of Gastrointestinal and Hepatobiliary Surgery, Department of Surgical Oncology, Tata Memorial Hospital, Homi Bhabha National Institute, Mumbai, Maharashtra, India

^b^Department of Biostatistics, Tata Memorial Hospital, Homi Bhabha National Institute, Mumbai, Maharashtra, India

^c^Department of Medical Oncology, Tata Memorial Hospital, Homi Bhabha National Institute, Mumbai, Maharashtra, India

**Corresponding author:**

Mahesh Goel
Professor, Chief Hepatobiliary Surgeon, 
Department of Surgical Oncology,
Tata Memorial Centre, Homi Bhabha National Institute
Ernest Borges Marg, Parel, Mumbai, 400012, India. 
E-Mail: [drmaheshgoel@gmail.com](mailto:drmaheshgoel@gmail.com)
Contact number: [+919820504492](mailto:drmaheshgoel@gmail.com)

**Supplementary Materials - Index**

| **Supplementary Methods** |  |
| --- | --- |
| Ethics approval and consent to participate | *Page 3* |
|  |  |
| **Supplementary Figures and Tables** |  |
|  |  |
| **Supplementary Table S1:** TMH criteria for Locally advanced/Borderline Resectable GBC used as an indication for Neoadjuvant Chemotherapy.  **Supplementary Figure S1:** Flow diagram depicting the inclusion process, methodology and exclusion, and treatment of GBC patients undergoing neoadjuvant chemotherapy.  (GBC: Gallbladder cancer; NACT: neoadjuvant chemotherapy; CBR: Clinical Benefit Rate) | *Page 4*  *Page 5* |
|  |  |
|  |  |

**Supplementary Methods**

**Ethics approval and consent to participate**

The data of the present study were collected in the course of common clinical practice, and accordingly, the signed informed consent was obtained from each patient for any surgical and clinical procedure. The study protocol was in accordance with the ethical standards of the institutional research committee and the 1964 Helsinki Declaration and its later amendments.

As this was a retrospective study, formal consent for this study is not required and no approval of the institutional research committee was needed.

**Supplementary Table and Figure**

| **Tumour characteristics** | | |
| --- | --- | --- |
| **Per primum GBC** | **Tumour**  **(T3–T4 tumours)** | Contiguous liver involvement > 2 cm |
|  |  | Involvement of bile duct causing obstructive jaundice (Type I/II block on MRCP/ERCP/PTBD) |
|  |  | Radiological/Endoscopic involvement of antropyloric region of stomach, duodenum, hepatic flexure of colon or small intestine |
|  | **Node**  **(N1 station)** | Radiological suspicion of lymph node involvement N1   - Hepatic artery (Station 8) - Hepatoduodenal ligament (Station12), - Retro pancreatic/retroduodenal (Station 13) Size > 1 cm in short axis, round in shape, and heterogenous enhancement on CT/PET scan. |
|  | **Vascular**  **(T4 tumours)** | Impingement/involvement (<180-degree angle) of one or more of the following blood vessels:   - Common Hepatic Artery and Right & Left Hepatic artery - Main Portal vein and Right & Left Portal vein |
| **For incidental GBC** | | Residual/Recurrent mass in GB fossa/liver bed |
|  |  | N1 nodes as per nodal criteria. |
|  |  | Involvement of bile duct causing OJ (Type I/II Block) |

**Supplementary Table S1:** TMH criteria for Locally advanced/Borderline Resectable GBC used as an indication for Neoadjuvant Chemotherapy

(T: Tumour stage, N: Nodal stage, MRCP: Magnetic Resonance Cholangiopancreatography, ERCP: Endoscopic Retrograde Cholangiopancreatography, PTBD: Percutaneous Transhepatic Biliary Drainage, CT: Computerised Tomography, PET: Positron Emission Tomography, GB: Gallbladder)

**
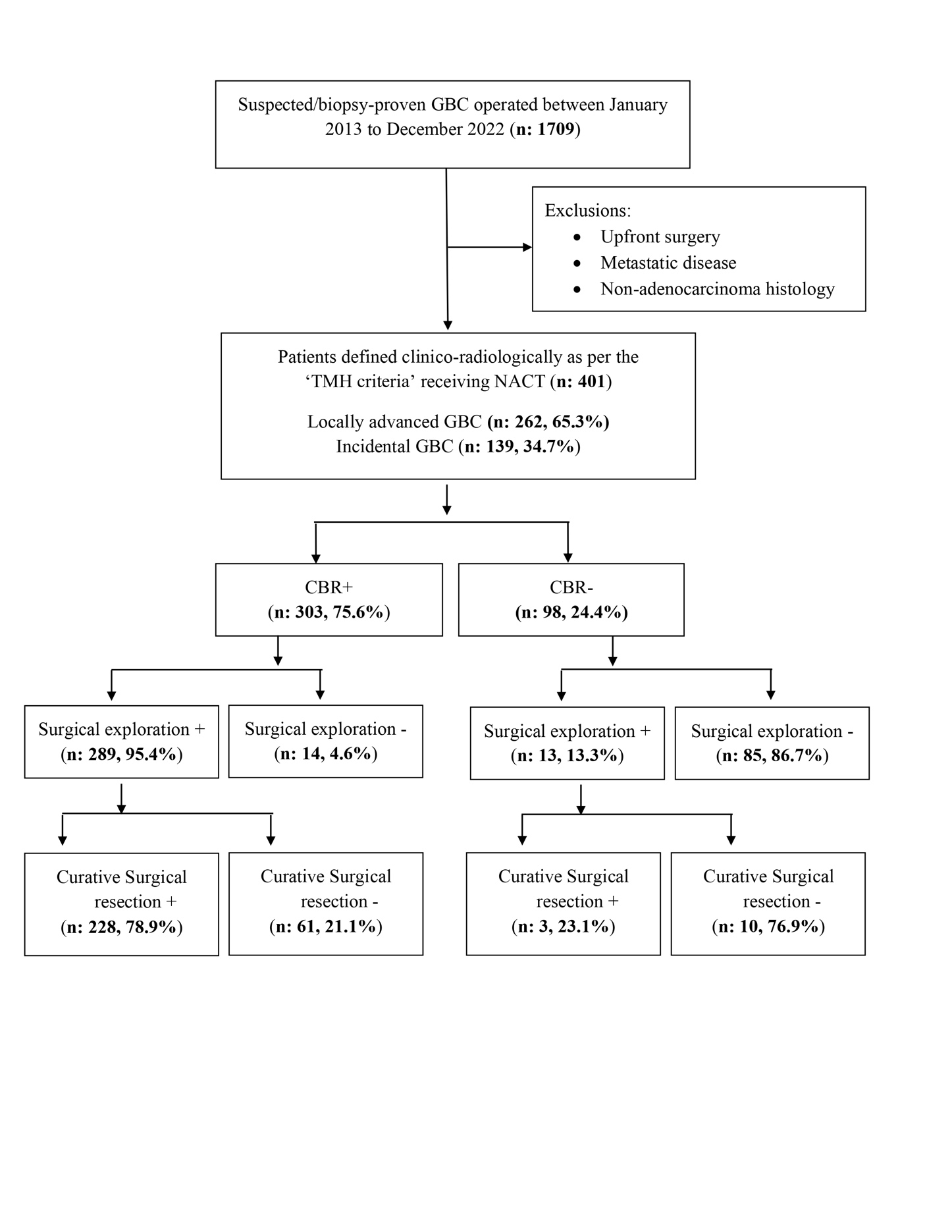
**

**Supplementary Figure S1:** Flow diagram depicting the inclusion process, methodology and exclusion, and treatment of GBC patients undergoing neoadjuvant chemotherapy.

(GBC: Gallbladder cancer; NACT: neoadjuvant chemotherapy; CBR: Clinical Benefit Rate)
